# metPropagate: network-guided propagation of metabolomic information for prioritization of metabolic disease genes

**DOI:** 10.1038/s41525-020-0132-5

**Published:** 2020-07-02

**Authors:** Emma J. Graham Linck, Phillip A. Richmond, Maja Tarailo-Graovac, Udo Engelke, Leo A. J. Kluijtmans, Karlien L. M. Coene, Ron A. Wevers, Wyeth Wasserman, Clara D. M. van Karnebeek, Sara Mostafavi

**Affiliations:** 1 BC Children’s Hospital Research Institute, Centre for Molecular Medicine and Therapeutics, University of British Columbia, Vancouver, Canada; 2 Departments of Biochemistry, Molecular Biology and Medical Genetics, Cumming School of Medicine, University of Calgary, Calgary, Canada; 3 Alberta Children’s Hospital Research Institute, University of Calgary, Calgary, Canada; 4 Translational Metabolic Laboratory, Department of Laboratory Medicine, Radboud University Medical Center, Nijmegen, The Netherlands; 5 Department of Medical Genetics, University of British Columbia, Vancouver, Canada; 6 Department of Pediatrics, BC Children’s Hospital Research Institute, Centre for Molecular Medicine and Therapeutics, University of British Columbia, Vancouver, Canada; 7 Department of Pediatrics, Radboud University Medical Center, Nijmegen, The Netherlands; 8 Department of Statistics, University of British Columbia, Vancouver, Canada

**Keywords:** Bioinformatics, Gene regulatory networks, Metabolic disorders, Medical genetics

## Abstract

Many inborn errors of metabolism (IEMs) are amenable to treatment, therefore early diagnosis is imperative. Whole-exome sequencing (WES) variant prioritization coupled with phenotype-guided clinical and bioinformatics expertise is typically used to identify disease-causing variants; however, it can be challenging to identify the causal candidate gene when a large number of rare and potentially pathogenic variants are detected. Here, we present a network-based approach, metPropagate, that uses untargeted metabolomics (UM) data from a single patient and a group of controls to prioritize candidate genes in patients with suspected IEMs. We validate metPropagate on 107 patients with IEMs diagnosed in Miller et al. (2015) and 11 patients with both CNS and metabolic abnormalities. The metPropagate method ranks candidate genes by label propagation, a graph-smoothing algorithm that considers each gene’s metabolic perturbation in addition to the network of interactions between neighbors. metPropagate was able to prioritize at least one causative gene in the top 20^th^ percentile of candidate genes for 92% of patients with known IEMs. Applied to patients with suspected neurometabolic disease, metPropagate placed at least one causative gene in the top 20^th^ percentile in 9/11 patients, and ranked the causative gene more highly than Exomiser’s phenotype-based ranking in 6/11 patients. Interestingly, ranking by a weighted combination of metPropagate and Exomiser scores resulted in improved prioritization. The results of this study indicate that network-based analysis of UM data can provide an additional mode of evidence to prioritize causal genes in patients with suspected IEMs.

## Introduction

Inborn errors of metabolism (IEMs) are the largest group of genetic diseases amenable to therapy, and are defined as any condition that leads to a disruption of a metabolic pathway, irrespective of whether it is associated with an abnormal biochemical test^1^. A growing understanding of metabolic and genetic phenotypes has resulted in the identification of at least 1015 well-characterized IEMs^1^. Identifying the causal gene has in turn provided insights and opportunities for interventions targeting downstream molecular or cellular abnormalities^2–4^. These efforts have been catalogued in the online resource IEMbase, which provides further information on the etiology and treatment of over 500 IEMs^5^. Early detection of IEMs, for example in the general population through newborn metabolic screening programs or in disease cohorts via genetics profiling, is pivotal so that treatment can be initiated before the onset of irreversible progressive organ damage. Without rapid treatment, damage to the central nervous system due to an IEM can result in intellectual disability disorder (IDD).

Identifying the genetic basis of IEMs has typically been performed using WES coupled with frequency and pathogenicity-guided variant prioritization. The promise of this approach was illustrated by a gene discovery study in which deep phenotyping and WES of patients with unexplained neurometabolic phenotypes achieved a diagnostic yield of 68%, identifying novel human disease genes and most importantly enabling targeted interventions in 44% of patients^6^. More broadly, published studies applying WES coupled with variant prioritization in patients with unexplained phenotypes are successful in identifying the underlying cause in 16 to 68% of patients^6^.

The significant time and reasoning required to identify the causative gene after WES analysis has led to the development of a variety of variant prioritization algorithms. These approaches take phenotype-specific and variant-specific characteristics into consideration to prioritize patient-specific candidate genes. One approach, CIPHER, uses networks of human protein–protein interactions, disease phenotype similarities, and known gene–phenotype associations to predict and prioritize disease genes^7^. Exomiser’s hiPHIVE algorithm maps human phenotype ontology terms across species, enabling researchers to leverage databases of well-phenotyped single-gene knock out animal models to identify the causative gene in a single patient^8^. These approaches have demonstrated the utility of protein–protein and protein–phenotype interaction networks in prioritizing candidate genes.

However, prioritized variants—even in the case of a fitting gene-phenotype match—often have a low level of supporting evidence for causality, and are thus not adequate to establish a genetic-based diagnosis. Using multiple types of personalized “-omic” data is a promising approach to address the evidence gap in support of an IEM diagnosis. While RNA profiling has been popular, it still leaves much to be desired with regards to diagnoses^9^. For patients suspected to have a metabolic disorder, integration of metabolomics data with WES/WGS data can provide direct evidence of a gene’s causality based on its implied metabolic dysfunction. For example, detection of the metabolite N-acetylmannosamine in cerebrospinal fluid can lend support to the identification of *NANS* as a causal gene^4^. These biochemical biomarkers can be detected individually (targeted metabolomics), or as part of a broader characterization of the metabolome (untargeted metabolomics).

**Fig. 1:**
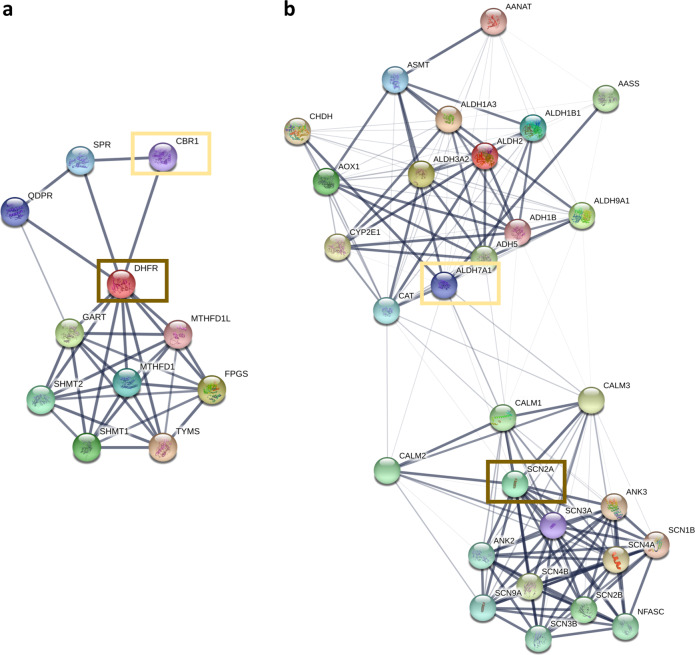
STRING network connectivity of two genes, *DHFR* and *SCN2A*. **a** DHFR (brown box) is connected to genes involved in folate biosynthesis in the STRING network (stringdb.org)^12^. Perturbation of metabolites associated with genes in *DHFR’s* neighborhood interaction partners, such as *CBR1* (yellow box), could be used to implicate its metabolic dysregulation. **b** *SCN2A* (brown box) is connected to the SCN family of ion channel proteins through co-expression analyses, and connected to another cluster of genes involved in monoamine metabolite biosynthesis, including *ALDH7A1* (yellow box), through co-citation in PubMed abstracts. Therefore, perturbed monoamine metabolite biosynthesis, despite being unrelated to the function of *SCN2A*, could point to *SCN2A* as the causal gene.

Recently, two separate untargeted metabolomics analysis pipelines were able to measure metabolites diagnostic for 20 of 21 and 42 of 46 IEMs, respectively^10,11^. These studies demonstrate that untargeted metabolomics analyses are able to measure clinically relevant metabolic phenotypes. However, it is important to note that available chromatographic and MS acquisition technologies are not able to measure all metabolites in a single individual, even when used in combination. This means that, depending on the combination of metabolomic systems used, some portion of metabolites will be missed, and the analysis will be insensitive to diseases associated with these unmeasured metabolites. Protein–protein interactions networks offer a potential solution to this problem, as they allow a perturbation in metabolites associated with Gene A to implicate Gene B. For instance, *DHFR* (dihydrofolate reductase), in the absence of folate differential abundance, could be prioritized by the detection of metabolites associated with one of its interaction partners, *CBL* (carbonyl reductase), which it is connected to through shared membership in the folate biosynthesis KEGG pathway (Fig. 1a). This method could also be used to prioritize non-IEM genes. For example, *SCN2A*, which is not associated with any metabolites detectable by LC/MS, could be prioritized by detection of differentially abundant metabolites (DAMs) associated with the *ALDH7A1* gene (Fig. 1b). In this case, given the lack of physical interaction between *SCN2A* and *ALDH7A1*, their connection represents their frequent co-citation, attributable to their common relationship to epilepsy. These examples illustrate how understanding how proteins interact with each other and their shared functions may help implicate (1) genes with metabolites that are undetectable by a given metabolomic system and (2) non-metabolic genes that share disease or pathway-level associations with metabolic genes.

In this paper, we describe a gene prioritization approach, called metPropagate, that uses patient-specific metabolomic data to prioritize candidate genes in patients with suspected metabolic disorders. metPropagate uses untargeted metabolomic data and gene-metabolite interaction databases to identify proteins whose metabolic function are perturbed in a given patient. We then use this information to assign a score to each protein in a protein functional linkage network (STRING), and then propagate this evidence across the network^12^. Each patient’s set of candidate genes is ranked by the resulting propagated score, resulting in a prioritized list of candidate genes. We apply this method to both curated and non-curated untargeted metabolomic datasets. Our curated dataset, which was initially described by Miller and colleagues (hereby referred to as the Miller data set), consists of 107 patients with diagnosed IEMs, each with a confirmed set of metabolite intensities from high-resolution untargeted LC/MS (Orbitrap) and GC/MS (Trace-DSQ) metabolomic analyses^10^. To determine the utility of metPropagate when applied to non-curated LC-MS metabolomic data, we also applied metPropagate to 11 patients diagnosed through the TIDEX neurometabolic discovery project at BC Children’s Hospital at the University of British Columbia. Notably, these two datasets differed in the types of diagnoses made. All patients in the Miller data set were diagnosed with an IEM; in the TIDEX data set, in contrast, all patients were suspected to have an IEM at time of study enrollment, but the majority were diagnosed with a neurogenetic disease that included abnormal metabolic characteristics. Application of metPropagate to these two contexts represents its flexibility in prioritizing genes causing both classic IEMs and neurogenetic disorders with metabolic phenotypes.

We show here that metPropagate is able to prioritize candidate genes from Exomiser’s variant filtering pipeline at a similar rate to Exomiser’s Human Phenotype Ontology term-driven prioritization algorithm^13^. Interestingly, we found that metPropagate and Exomiser’s phenotype-driven algorithm complement each other, as causative genes prioritized by one algorithm were often not prioritized by the other. Additionally, ranking by a weighted combination of metPropagate and Exomiser scores resulted in prioritization of at least one causative gene in 10/11 patients, an improvement over either algorithm alone. This paper demonstrates that curated and non-curated untargeted metabolomic data from a single patient can be used in conjunction with protein–protein interaction networks to prioritize causative genes by providing an additional stream of evidence of a gene’s impaired function. This prioritization technique can be used to complement existing variant-based and phenotype-based prioritization algorithms.

## Results

**Fig. 2:**
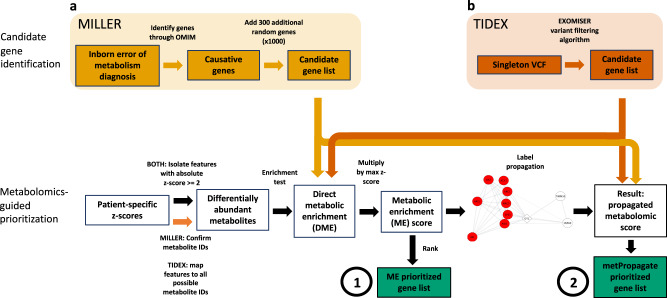
Analysis overview. **a** For each patient in the Miller dataset, 1000 permutations of random candidate gene lists that included the causative gene were generated. Differentially abundant metabolites (DAMs) were identified as those with an absolute z-score greater than or equal to 2. The per-gene enrichment *p*-value and absolute value of the largest metabolite z-score annotated to each gene were multiplied, generating a per-gene metabolic enrichment (ME) score, which was used to rank candidate genes (circle 1). For the metPropagate analysis, the DME scores were scaled between 0 and 1 and used as weights assigned to each gene in the STRING network. Label propagation generated a score for each gene that could be used to identify median rank of causative gene across all permutations (circle 2). **b** TIDEX: The causative gene in each patient was previously identified^6^. Singleton VCFs for all patient were created by DeepVariant, which were then filtered by Exomiser to generate a list of candidate genes. Raw LC/MS files for a single patient and a group of biofluid-specific controls were analyzed using XCMS. Samples were normalized and z-scores were generated for each feature using biofluid-specific controls as reference. Features were mapped to metabolites using HMDB. DME and metPropagate scores were then calculated as in (**a**).

The metPropagate algorithm uses patient-specific untargeted metabolomic data (both curated and non-curated) to prioritize a list of candidate genes. In this paper, curated metabolomic data is untargeted data that has been subset to only include intensities of metabolites that have a confirmed identity; in contrast, non-curated metabolomic data is untargeted data in which a *m/z* ratio can represent multiple different metabolites (e.g., the intensity of a feature with *m/z* 200 could be included in the data set as 10 different metabolites). This list of candidate genes can originate through a WES or WGS filtering pipeline, or be chosen *a priori*. In this study, we first demonstrate metPropagate’s applicability to the Miller data set, a previously published, curated untargeted LC/MS and GC/MS metabolomics data set consisting of 107 patients with IEMs (Fig. 2a). We then apply metPropagate to a non-curated untargeted LC/MS dataset consisting of eleven patients diagnosed with neurometabolic disease through the TIDEX project (Fig. 2b). Neurometabolic disease was defined as the presence of (1) CNS and (2) metabolic abnormalities (see “Methods” section). Importantly, although all TIDEX patients exhibited metabolic abnormalities at time of study enrollment, the majority of patients were not diagnosed with an inborn error of metabolism, highlighting metPropagate’s utility in diagnosing neurogenetic diseases with some metabolic features. We compare metPropagate to currently used metabolomics-based prioritization methods, in addition to a clinical phenotype-driven prioritization algorithm: Exomiser’s phenotype score in the hiPHIVE algorithm^13^.

### Gene-based metabolomic enrichment tests can prioritize a causative gene when annotated metabolites are differentially abundant

**Fig. 3:**
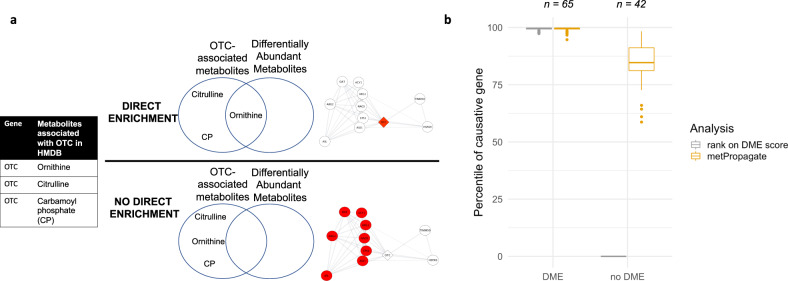
Prioritization of IEM-related genes using Miller et al. curated, untargeted metabolomics data. **a** Gene-metabolite associations were extracted from HMDB. Genes that were deemed to have differential metabolic enrichment (DME) have associated metabolites that are differentially abundant, and therefore have an ME score by which they can be ranked. Genes that do not have DME either do not have any metabolites that are differentially abundant or have metabolites that are undetectable by a given metabolomic system (e.g., sodium, ATP). As an example, the gene *OTC* has direct enrichment for citrulline, ornithine, and carbamoyl phosphate. DME is possible when one of these metabolites is differentially abundant. However, when these three metabolites are not differentially abundant (typically due to measurement limitations), metPropagate theoretically has the capacity to prioritize the *OTC* based on DME of other genes in its local neighborhood. **b** Boxplot of percentile rank of causative gene after ranking with DME and metPropagate algorithms. If the causative gene exhibited DME, the causative gene was prioritized in the top 20^th^ percentile with both the ME and metPropagate algorithms. If the causative gene did not exhibit DME, the ME algorithm failed to prioritize the causative gene in all cases, but metPropagate was able to prioritize the causative gene in 79% of cases.

The Miller data set consists of 436 plasma metabolite z-scores for 107 patients diagnosed with one of 21 IEMs (Table 1). Each z-score was generated by comparing the intensity of a given metabolite in a patient with the intensity of that metabolite in a group of 75 controls. Importantly, no genetic information other than each patient’s IEM diagnosis was made available in the Miller et al. publication. We sought to determine if metabolomic data and gene-metabolite associations available in the Human Metabolome Database (HMDB) could be used to prioritize at least one gene associated with each IEM in OMIM (Fig. 2a, Table 1)^14^. The HMDB database is routinely used to annotate m/z features in untargeted metabolomic experiments due to the large number of annotated metabolites and detailed isotope, adduct and structural information available (Fig. 3a)^11^. To identify genes that may be causing perturbations in a single patient’s metabolome, metabolites with significantly higher or lower abundance than in controls, hereby referred to as DAMs, were mapped to HMDB genes, and statistical enrichment of DAMs among each group of gene-associated metabolites was assessed using a Fisher’s Exact Test. To generate a score that could be used to rank candidate genes, we multiped the scaled enrichment *p*-value (differentially metabolic enrichment—DME) by the absolute value of each gene’s largest metabolite z-score, generating a per-gene score called the metabolic enrichment (ME) score. For each patient, we calculated the median rank of the ME score of the causative gene(s) across 1000 permuted sets of 300 randomly chosen genes. We delineated our results by causative genes that had an ME score (i.e., metabolites associated with the causative gene were differentially abundant) and those that did not. At least one causative gene had an ME score in 61% of patients; in these cases, the causative gene was prioritized in the top 20^th^ percentile (Fig. 2a, circle 1; Fig. 3a, b). Causative genes with an ME score of zero were not able to be prioritized. Notably, metabolites commonly used as IEM biomarkers, such as acyl glycines or acyl carnitines, are not annotated to IEM-associated genes in HMDB (e.g., isovalerylglycine, which is a biomarker of 3-methylcrotonyl-CoA carboxylase deficiency, is annotated to the *GLYAT* family of genes, but not to either of the IEM-associated genes *MCCC1* or *MCCC2*). Further, metabolites profiled in the Miller data set mapped to only 37.3% of all genes listed in HMDB. This suggests that using the ME score to directly rank candidate genes lacks the sensitivity needed to reliably identify causative genes.

**Table 1:** Miller IEMs and associated genes.

Inborn error of metabolism	Gene names
3-methylcrotonyl CoA carboxylase deficiency	MCCC1, MCCC2
Argininosuccinic acid lyase deficiency	ASL
Argininemia	ARG1
Cobalamin biosynthesis deficiency	MUT, MTR, MTRR, MMADHC, MMAB, MMACHC, MMAA, LMBRD1
Citrullinemia	ASS1, SLC25A13
Glutaric Aciduria type 1	GCDH
Glutaric Aciduria type 2	ETFA, ETFDH, ETFB
3-OH-3methylglutaryl (HMG) CoA lyase deficiency	HMGCL
Holocarboxylase deficiency	HLCS
Homocystinuria	MTHFR, MTRR, MTR, MMADHC
Isovaleric aciduria	IVD
Lysinuric protein intolerance	SLC7A7
Medium chain acyl-CoA dehydrogenase deficiency	ACADM
Methylmalonic aciduria	MCEE, MMADHC, MMAB, MMAA
Maple syrup urine disease	BCKDHB, BCKDHA, DBT
Ornithine transcarbamoylase deficiency	OTC
Propionic aciduria	PCCB, PCCA
Phenylketonuria	PAH
Thymidine Phosphorylase deficiency	TYMP
Trimethyllysine hydroxylase epsilon deficiency	TMLHE
Very-long chain acyl-CoA dehydrogenase deficiency	ACADVL

### metPropagate can prioritize causative metabolic genes regardless of metabolic enrichment status of causative gene

**Fig. 4:**
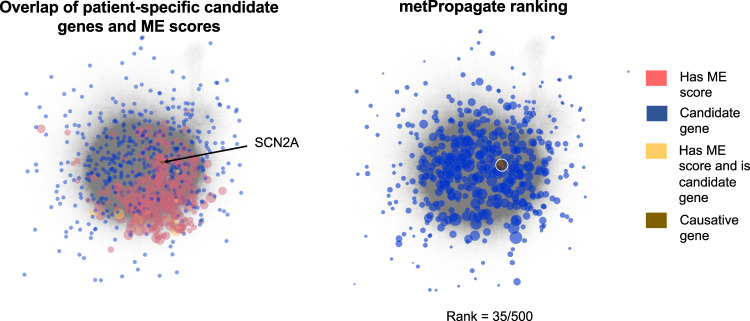
Overlay of initial scores, Exomiser-Phenotype and metPropagate rankings of all candidate genes from a single TIDEX patient with a causative mutation in *SCN2A*. In the panel on the left, genes that were found to have an ME score are red, genes that were identified as candidates by Exomiser’s variant filtering pipeline are blue, and genes that are both candidate genes and have DME are yellow. The causative gene is brown. The size of the node corresponds to its ME score. In the panel on the right, the size of the gene is proportional to the metPropagate score after label propagation. In this particular patient, although the causative gene (*SCN2A*) was not directly metabolically enriched, *SCN2A* received some metabolomic signal from its enriched neighbors through label propagation, facilitating its prioritization.

We applied metPropagate to the Miller data set to determine whether metPropagate could prioritize IEM-related causative genes, particularly those that were not prioritized via ME score. metPropagate expands the number of prioritizable genes by propagating per-gene ME scores across a protein–protein functional linkage network (Fig. 4). metPropagate outputs a score for each gene in the network that summarizes the degree to which that protein’s neighborhood was enriched for DAMs. Among patients with causative genes that did not have an ME score, metPropagate was able to prioritize one causative gene in the top 20^th^ percentile for 79% of patients (33/42) (Fig. 3b). Out of all 107 patients, metPropagate was able to prioritize the causative gene in 92% (98/107) of patients, 31% (33/107) more than with the ME score alone. The rank of each candidate gene across all permutations is provided in Supplementary Data Set 1. These results indicate that metPropagate is able to prioritize genes even when metabolites associated with the causative gene are not observed or detected.

### metPropagate can prioritize causative genes in patients with neurometabolic disease

**Fig. 5:**
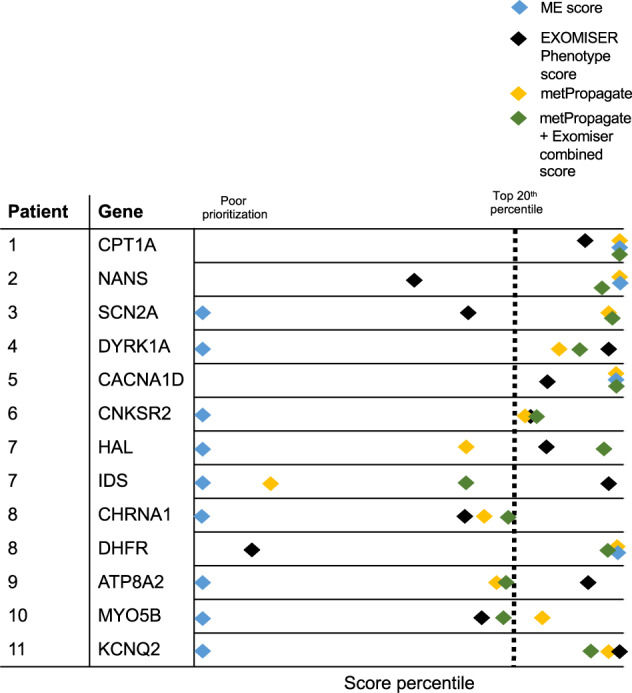
metPropagate prioritization in the TIDEX dataset. Relative percentile prioritization of candidate gene with metPropagate (gold), Exomiser’s phenotype score (black), metabolic enrichment score (blue) and combined metPropagate and Exomiser score (green). The ME algorithm places the causative gene in the top 20^th^ percentile in only 4/11 patients (blue), as the causative genes in the remaining patients did not exhibit DME. metPropagate places the causative gene in the top 20^th^ percentile of candidates in 9/11 patients. Exomiser-Phenotype places the causative gene in the top 20^th^ percentile of candidates in 7/11 of patients. The combined metPropagate + Exomiser-Phenotype score places the causative gene in the top 20^th^ percentile of candidates in 10/11 patients.

Next, we wanted to determine whether metPropagate could prioritize the causative gene from a list of WES-derived candidate genes using untargeted metabolomic data with unconfirmed metabolite identities (Fig. 2b, circle 1 and 2). We applied metPropagate to 11 patients who had been previously genetically diagnosed through the TIDEX neurometabolic gene discovery project^6^. Overall, one causative gene was identified in 9 patients and two causative genes were identified in 2 patients, resulting in a total of 13 causative genes. For each patient, between 281 and 609 total genes emerged from Exomiser’s variant effect and population frequency filters (Table 2). Although all patients were suspected to have neurometabolic disease caused by an IEM at time of enrollment, 5/11 patients were found to have mutations in a known IEM-causing gene (*CPT1A, NANS, HAL/IDS, ATP8A2, DHFR*), and 7/11 patients were found to have mutations in known neurogenetic disease genes (*SCN2A, CACNA1D, CNKSR2, MYO5B, KCNQ2, CHRNA1, DYRK1A*). Therefore, application of metPropagate to this patient population served to determine whether metabolomic data could be used to prioritize both IEM genes and non-IEM genes. Each patient’s metabolomic features, defined by *m/z* ratio and intensity values, were derived from untargeted metabolomics data and compared to a group of controls (separate controls for CSF and plasma) to generate a z-score for each feature. Due to lack of availability of feature to metabolite mapping for the metabolomic system used in the TIDEX study, we were unable to identify the exact metabolic identify of each metabolic feature. Therefore, features were matched to all possible metabolite identities using HMDB through exact-mass matching. All possible metabolite IDs were retained for the enrichment analysis. To illustrate how metPropagate improves prioritization, a visual representation of the overlap between candidate genes, metabolic enrichment scores, and metPropagate scores for a patient with an *SCN2A* mutation is provided in Fig. 4. More generally, metPropagate prioritized at least one causative gene in the top 20^th^ percentile of candidate genes in 9/11 patients (9/13 genes), in the top 10th percentile in 6/11 patients (6/13 genes) and in the top 5th percentile in 5/11 patients (5/13 genes) (Fig. 5). We sought to compare this prioritization to that achievable using other prioritization methods: ME and Exomizer’s hiPHIVE phenotype score. Using ME, the causative gene was prioritized in the top 20^th^ percentile in 4/11 patients, three of whom had mutations in known IEM genes. Notably, although *IDS* mutation does cause an IEM, the metabolic system used was not able to measure the abundance of large glycoaminoglycans, highlighting a limitation of ME analysis. metPropagate prioritized the causative gene in more patients than clinical phenotype-driven component of Exomiser’s hiPHIVE algorithm (Exomiser-Phenotype) (Table 2). Exomiser-Phenotype placed the causative gene in the top 20^th^ percentile in 7/11 patients (8/13 genes), in the top 10^th^ percentile in 4/11 patients (5/13 genes), and in the top 5^th^ percentile in 3/11 patients (4/13 genes). Exomiser-Phenotype’s ranking of the causative gene was higher in 5/11 patients, and 7/13 genes. metPropagate prioritized the causative gene in all patients prioritized by ME score. Interestingly, metPropagate and Exomiser-Phenotype prioritized the causative gene in 5/7 and 4/7 of patients the ME score failed to prioritize, respectively. Further, at least one algorithm prioritized the causative gene in each of the eleven patients. These results suggest that metPropagate outperforms prioritization by ME, and may complement existing phenotype-driven approaches to prioritization.

**Table 2:** Comparison of raw ranking between metPropagate, Exomiser’s phenotype score, the weighted metPropagate + Exomiser score and ME score.

Patient	Gene	IEM status	Biofluid analyzed	Number of candidate genes	Metabolic enrichment (ME)	metPropagate	Exomiser’s phenotype score	Weighted metPropagate + Exomiser score
1	CPT1A	IEM	CSF	579	1/15*	2*	26*	1*
2	NANS	IEM	CSF	390	5/7*	5*	207	12*
3	SCN2A	Non-IEM	CSF	500	NA	35*	132	26*
4	DYRK1A	Non-IEM	CSF	281	NA	38*	14*	27*
5	CACNA1D	Non-IEM	Plasma	378	1/23*	1*	52*	1*
6	CNKSR2	Non-IEM	Plasma	443	NA	75*	59*	50*
7	HAL	IEM	Plasma	383	NA	128	60*	18*
7	IDS	IEM	Plasma	383	NA	252	20*	99
8	CHRNA1	Non-IEM	Plasma	520	NA	153	143	84*
8	DHFR	IEM	Plasma	520	9/17*	7*	293	46*
9	ATP8A2	Non-IEM	CSF	609	NA	136	28*	73*
10	MYO5B	Non-IEM	CSF	395	NA	65*	123	85
11	KCNQ2	Non-IEM	CSF	350	NA	15*	3*	24*

Asterix (*) indicates prioritization in the top 20^th^ percentile of candidate genes.

### metPropagate ranking is affected by gene and patient-specific characteristics

**Fig. 6:**
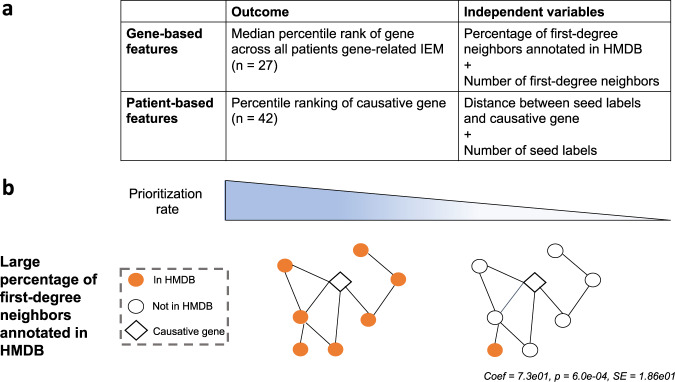
Gene-based and patient-based factors that affect prioritization at the gene and patient metabolome level. **a** This analysis only included patients with causative genes that did not exhibit DME (*n* = 42). Separately, generalized linear models were used to assess the relationship between a gene’s median rank and the number of first-degree neighbors and percentage of them annotated in HMDB, and the relationship between the percentile ranking of an individual’s causative gene and the number of and distance between seed labels. **b** Genes with large percentage of first-degree neighbors annotated in HMDB have a higher prioritization rate. The number of seed labels, as well as the distance between the causative gene and seed labels, did not significantly impact the rank of the causative gene.

As the output of any network-based algorithm depends on network connectivity, we next aimed to identify gene-specific and patient-specific factors that affect gene prioritization. In order to identify factors that affect a gene’s prioritization by metPropagate, we used the Miller data set to collect patient-specific and causative gene-specific characteristics and correlated these variables with metPropagate’s percentile ranking of the causative gene. We confined our analysis to patients who had exhibited no ME in the causative gene(s), as we wanted to ensure that any gene-specific or patient-specific characteristic that influenced the ranking of the causative gene could be tied to the causative gene’s neighborhood. The information gathered on each patient included characteristics of their seed ME profile: the average distance between seed genes (initial labels) and the causative gene in the STRING network, and the number of metabolically enriched genes. Neither the number of enriched genes nor the median distance between a patient’s causative gene and its initial labels was positively associated with the percentile rank of the causative gene(s). For each gene, we gathered information on the number of first-degree neighbors and the percentage of first-degree neighbors annotated in HMDB (Fig. 6). We found that both the number and percentage of HMDB-annotated first-degree neighbors (hereby referred to as metabolic first-degree neighbors) were independently positively associated with that gene’s median percentile ranking across all patients with the same IEM (*p* = 2.9e−09, coef = 6.5e−02, SE = 7.1e−03; *p* = 6.0e−04, coef = 73.384890, SE = 18.594193, *n* = 27).

The relationship between gene prioritization and node degree has been found by others applying label propagation algorithms to diverse networks, and is not surprising given the understanding that genes that have multiple functionalities will be more affected by metabolic abnormalities in diverse biological pathways^15^. Similarly, it makes sense that genes that interact with metabolic genes have a higher likelihood of being prioritized by a metabolomics-driven prioritization algorithm. However, this association may lead genes with a large HMDB neighborhood to be more vulnerable to false positive metabolic enrichment; indeed, across all 19,354 genes in 107 patients, we find a positive correlation between percentage of metabolic first-degree neighbors and a gene’s median percentile ranking. Given these findings, we next decided to build a model that combined metPropagate and Exomiser-Phenotype rankings in order to reduce the likelihood of false-positive prioritization.

### Combining Exomiser-Phenotype and metPropagate ranking increases prioritization rate of causative gene

Both metPropagate and Exomiser-Phenotype algorithms are vulnerable to false positive prioritization. metPropagate is more likely to prioritize genes with a high percentage of metabolic first-degree neighbors, and Exomiser-Phenotype is more likely to prioritize well-characterized disease genes^13^. We sought to combine metPropagate and Exomiser in a way that moderated metPropagate’s inherent bias, leading to improved prioritization. To do this, we created a weighted additive model that penalized metPropagate by the prior probability that a gene with a particular percentage of metabolic first neighbors would be prioritized in the top 20^th^ percentile (see “Methods” section). Effectively, this meant that the metPropagate ranking was weighted more heavily for genes with few metabolic first-degree neighbors, and the Exomiser ranking was weighted more heavily for genes with a high percentage of metabolic first-degree neighbors. We found that the combined metPropagate and Exomiser score prioritized the causative gene in the top 20^th^ percentile in 10/11 patients (11/13 genes), the top 10^th^ percentile in 8/11 patients (8/13 genes) and the top 5^th^ percentile in 4/11 patients. As shown in Fig. 5, the combined ranking was higher than either metPropagate’s or Exomiser’s rankings for 8/11 patients (9/13 genes), suggesting that the combined score reduces false positive prioritization. This indicates that when applied to a single patient, using metPropagate and Exomiser in conjunction can increase the likelihood of prioritizing the causative gene.

## Discussion

In this paper, we present metPropagate, an algorithm that uses a protein-protein functional interaction network and metabolomic information to prioritize candidate genes in patients with suspected IEMs. Rather than relying solely on detecting perturbed gene-associated metabolites, metPropagate uses evidence of functional interactions between proteins to be able to also prioritize candidate genes that either do not interact with any metabolites or interact with metabolites that are not measured in a given metabolomic system. metPropagate was able to use untargeted metabolomic information to prioritize at least one IEM-related gene in 92% of patients diagnosed with one of 21 known IEMs, 30% more than was possible with DME analysis alone. A similar result was observed using metabolomic data of unconfirmed metabolite identities. Specifically, in a set of eleven patients with previously diagnosed neurometabolic disease, metPropagate was able to prioritize the causative gene in the top 20^th^ percentile of candidates in 9/11 of patients. We used a weighted additive approach to combine metPropagate and Exomiser rankings to reduce the likelihood of false positive prioritization, resulting in improved ranking in 8/11 patients, and highlighting the value in combining variant prioritization algorithms that use orthogonal prioritization modalities.

STRING was chosen as the functional linkage network due to its strong performance in benchmarking analyses^16^. Evidence sources for edges in the STRING network include curated knowledge from databases such as KEGG and BioCarta, shared gene context (e.g., shared homology and coevolution) analyses, in addition to co-expression or co-citation studies. Based on the available evidence, certain protein pairs can be predicted to interact physically or functionally (e.g., shared catalysis/reaction/pathway or cooperation in performing epigenetic modifications). Using edge weights that reflect both physical and functional evidence of interaction has benefits and drawbacks. A major benefit is the possibility of modeling interactions between proteins that may not involve direct binding, but do influence dynamics of a local neighborhood. However, drawbacks include the addition of noise, as metabolic information may be propagated between proteins that share a function, rather than a physical bond. Due to the pros and cons of both approaches, we decided to empirically determine whether subsetting STRING to include just physical interactions or both physical and functional interactions leads to improved prioritization. We found that including both functional and physical interactions in the STRING network resulted in increased prioritization, therefore the complete STRING was used in all analyses (see “Methods” section on propagating metabolomic seed labels).

In the application of metPropagate to the TIDEX study, singleton exomes were analyzed, resulting in long lists of candidate genes. This was done to simplify comparisons between Exomiser and metPropagate; however, in reality, trio WES is performed whenever parental samples are available, which simplifies prioritization and shortens the candidate gene list. In the current study, candidate gene lists for a single patient included between 281 and 609 genes, meaning that the top 20^th^ percentile included 55–115 genes. Reducing the number of candidate genes examined by incorporating parental genomes into the variant filtering process would improve the interpretability of prioritization results.

Many recent meta-studies of the efficacy of WES in diagnosing rare genetic diseases have revealed that its yield is usually less than 50%^17^. As such, for unsolved cases, researchers and clinicians are increasingly turning to WGS to identify candidate causal genes; however, the expansion into the rest of the genome causes a dramatic increase in the number of candidate variants. This is in part due to difficulties interpreting the effect of variants beyond the exome. As such, supplemental information regarding dysregulated candidate genes that can be obtained via epigenomic, proteomic and metabolomic information is crucial^18^. We envision applying the metPropagate approach to highlight sets of candidate genes which may be metabolically impacted, thereby helping to identify causal candidate variants in the noncoding regions of the genome.

In summary, propagating metabolomic enrichment data across a protein functional linkage networks is a novel approach for prioritizing candidate genes in the context of suspected genetic metabolic disease. It improves upon existing gene-based direct metabolic enrichment tests and exhibits comparable performance to existing phenotype-based prioritization tools such as Exomiser’s hiPHIVE phenotype algorithm. Combining metPropagate and Exomiser rankings resulted in improved prioritization, suggesting that metPropagate can complement orthogonal gene prioritization approaches. Expansion of gene to metabolite associations and the use of multiple types of metabolomics platforms may help expand the number and type of genes that can be prioritized through this method.

## Methods

In this section, we will describe the data sets used in this study, the pre-processing applied to each data set, and the metPropagate algorithm. A complete outline of the metPropagate analysis pipeline is provided (Fig. 2).

### Data

Blood plasma samples from 117 patients diagnosed with 21 different IEMs underwent three different types of untargeted metabolic screening: (1) gas chromatography coupled mass spectrometry (GC-MS), (2) liquid chromatography coupled mass spectrometry (LC-MS) in positive ion mode and (3) LC-MS in negative ion mode. GC-MS analysis was performed using a Trace DSQ fast-scanning single-quadruple mass spectrometer (Thermo-Finnigan), and LC-MS analysis performed using an Orbitrap Elite high-resolution mass spectrometer (Thermo-Finnigan). Raw analyte intensity values (features) were calculated as the area under the chromatographic peak. Features underwent median-scaling and missing value imputation with the minimum detected intensity value and filtering which removed any feature not found in at least 10% of all samples. Each feature was assigned a z-score based on the intensity of the feature relative to a control population of 70 non-IEM individuals (not age or sex matched). Features were mapped to metabolite identities using a library containing the chromatographic and spectral signatures of over 2500 metabolites originating from human metabolic processes. Full details of the analysis are provided in Miller et al.^10^. Only IEMs that were diagnosed in Miller et al. were included, reducing the total size of this data set to 107 patients (all patients with Guanidinoacetate methyltransferase deficiency were removed in Miller et al. due to lack of detected biomarkers). To simulate ranking of the causative gene from a list of candidates, 1000 permutations of 300-*n* random genes (where *n* is the number of possible causative genes annotated to a particular IEM, e.g., *MCCC1* and *MCCC2* for 3-methylcrotonyl CoA carboxylase deficiency) were selected from 19,354 genes annotated in STRING (v11, stringdb.org)^12^; for each patient, the median rank of each IEM-associated gene was recorded across all 107 patients (Supplementary Data Set 1). For analysis purposes, an IEM was considered prioritized when at least one IEM-associated causative gene was prioritized in the top 20^th^ percentile (i.e., rank is < =60).

This study also analyzed WES and LC/MS data from 11 patients genetically diagnosed through the TIDEX neurometabolic gene discovery project, hereby referred to as the “TIDEX project” (UBC IRB approval H12-00067) (Supplementary Table 1). Parents and caregivers provided written informed consent for the study. Details of this investigation have previously been published^6^ as well as in separate case reports^2–4,19,20^. Supplementary Table 1 summarizes the clinical characteristics of the patients and their previously identified genetic diagnoses. Patient inclusion criteria consisted of (1) a confirmed or potential neurodevelopmental disorder and (2) a metabolic phenotype. A metabolic phenotype could be reflected by (1) a pattern of abnormal metabolites in urine, blood or CSF, (2) abnormal results on biochemical functional studies or (3) typical abnormalities in clinical history or physical exam. Each individual in the TIDEX project underwent WES and untargeted metabolomic profiling of either CSF of plasma, depending on availability. WES analysis included data from the patients, their parents, and any other affected family members. DNA from unaffected members was used to confirm segregation with disease through Sanger analysis. Untargeted LC-MS metabolomic profiling was only performed on the proband.

### Whole-exome sequencing data processing

WES data from 11 patients meeting the aforementioned criteria was generated using the Agilent SureSelect capture kit and the Illumina HiSeq 2000 or 2500 sequencer. The WES data was filtered for variant frequency and quality using the pipeline described in Tarailo-Graovac et al.^6^. A team of bioinformaticians and medical geneticists then examined the resulting list of candidate genes and identified the causative variant(s) based on predicted pathogenicity of the causative variant as well as known disease/phenotype associations. Diagnoses were made for each of these patients prior to use within this study, integrating additional family members in a subset of the cases. For fairness of comparison across patients, the data was reprocessed using an updated pipeline as follows: read mapping with BWA mem (v. 0.7.5)^21^. Samtools for file format conversion (v. 1.3.0)^22^. Picard for duplicate read marking (v. 1.139) (http://broadinstitute.github.io/picard). GATK for indel realignment (v. 3.4-46)^23–25^ and DeepVariant (v. 0.8.0) for variant calling^26^. Owing to the improved accuracy of DeepVariant over previous methods, raw variant calls were not filtered using additional tools.

We used Exomiser’s (v. 11.0.0) variant filtering pipeline to identify a list of candidate genes for each of 11 patients analyzed through the TIDEX project. Singleton WES data from 11 patients was processed (described above) before being input to Exomiser, which applied variant frequency filters to remove common variants, annotated functional impact against genes and then categorized by inheritance pattern: autosomal recessive, autosomal dominant and mitochondrial. In this study, gene scores from all inheritance patterns were combined. In the case where a single-gene harbored variants of more than one inheritance pattern, the variant with the highest variant prioritization ranking was retained for further analysis. The resulting list of genes was considered the candidate gene list for that patient. To prioritize each gene, Exomiser’s hi-PHIVE phenotype algorithm (Exomiser-Phenotype) relied on user-specified patient-specific Human Phenotype Ontology (HPO) terms (v. 1807) to generate a “phenotype” score. HPO terms were generated for each patient manually based on deep clinical phenotyping write-ups. For genes that had been studied using a knockout mouse, zebrafish model, or that were known Mendelian disease genes, the phenotype score represented the similarity between the patient’s HPO terms and the mouse, zebrafish or human ontology terms associated with that knockout model or disease cohort. Additional information about the algorithm and databases used to calculate this phenotype score can be found in Smedley et al.^13^.

### TIDEX LC/MS metabolomics data generation and processing

High-resolution untargeted metabolomics analysis of CSF and plasma was performed using UHPLC-QTOF mass spectrometry. Due to sample availability, plasma was analyzed for four of the IEM patients and 10 of the controls, and CSF was analyzed for seven of the IEM patients and 15 of the controls. Only samples profiled in the same bio-fluid were compared. CSF and plasma samples were de-proteinized in methanol:ethanol solution (50:50; 100 microlitres of each sample plus 400 microlitres of methanol:ethanol solution). Samples were profiled in duplicate, however, only one of each duplicate pair was analyzed in this study. A 2-microlitre sample was applied to an Acquity HSS T3 reverse-phase column (100 mm × 2.1 mm; 100 Angstroms; 1.8 micrometer), and an Agilent 6540 UHD accurate mass UHPLC-QTOF mass spectrometer with acquisition in positive and negative modes was used. The buffers in positive mode consisted of buffer A (0.1% (v/v) formic acid in water) and buffer B (0.1% (v/v) formic acid in water:methanol solution (1:99)); in negative mode, the buffers consisted of buffer A (10 mM acetic acid) and buffer B (10 mM acetic acid in water:methanol solution (1:99))^11^.

Once MS data had been generated, the centwave and obiwarp methods in the XCMS package were used for peak detection and retention time correction, respectively, in both positive and negative electrospray ionization detection modes (v3.3.1)^27^. Data-driven parameters were optimized using the IPO package (v1.10.0)^28^, and are available at https://github.com/emmagraham/metPropagate/blob/master/XCMS_parameters.md. CAMERA was used to annotate adducts and isotopes (v1.36.0)^29^. Linear baseline normalization was applied to each feature^30^. In linear baseline normalization, a baseline intensity profile is created from the median intensity of all features across all samples (hereby referred to as “baseline”), and all runs are assumed to be scalar multiples of the baseline intensity profile. For each metabolite i in sample *j*:

1 $$y_{{\mathrm{i}}{\mathrm{j}}}^\prime = \beta _{\mathrm{j}}y_{{\mathrm{i}}{\mathrm{j}}}$$

Where *y’*_ij_ is the normalized abundance of a particular feature and *y*_ij_ is the log transformed unnormalized abundance. *β* is the per-sample scaling factor defined as the mean intensity of the baseline over the mean intensity of the sample (j):

2 $$\beta _{\mathrm{j}} = \frac{{{y_{{\mathrm{baseline}}}} }}{{\overline {y_{\mathrm{j}}} }}$$

Two filtering criteria were applied before analysis: removal of (1) features not annotated to any known metabolites in the HMDB and (2) features annotated as non-base isotopes^31^. Z-scores based on the mean and standard deviation of a given metabolite across all IEM patients and controls were computed. Features for which the IEM patient had a z-score greater than 2 (2 SD away from the mean) were isolated and called “differentially abundant metabolites” (DAMs). Metabolites with positive z-scores exhibited higher abundance than in controls, while metabolites with negative z-scores exhibited lower abundance than in controls. All DAMs found through both positive and negative mode analyses were annotated with compound identities within 15 ppm of the compound mass using HMDB. Results from both positive and negative modes were combined for subsequent enrichment tests.

### metPropagate

Though the steps described above, an LC-MS metabolomics pipeline identified DAMs and a gene-based analysis pipeline identified a set of candidate genes. The primary goal of subsequent analysis was to determine whether metabolomic evidence could be used to prioritize the causative gene from this list of candidate genes. To do this, each gene in a patient’s candidate gene list was ranked using a per-gene metabolomic score termed the “metPropagate score”, which represented the likely metabolic relevance of a particular gene to each patient. Per-gene metPropagate scores were generated by initializing a protein–protein functional linkage network with a metabolic “enrichment” score for each gene and propagating this score across the STRING network using a network propagation algorithm.

#### Calculating the metabolic enrichment score

A Fisher’s Exact enrichment test was performed to determine whether metabolites known to be associated with candidate genes were overrepresented in the patient-specific set of DAMs. Curated sets of metabolites associated with each putative gene were parsed from files available from the HMDB web portal (hmdb.ca, April 1st 2019)^31^. Enrichment was calculated using Fisher’s Exact test. *P*-values were adjusted for multiple testing using the Benjamini-Hochberg procedure, and reported as false discovery rate (FDR).

The metabolomic enrichment (ME) score was computed as follows:

3 $${\mathrm{ME}} = - {\mathrm{log2}}\left( {2 + p} \right) \ast Z$$

where p is the unadjusted enrichment *p*-value, and *Z* is the z-score of the largest magnitude of any metabolite annotated to that gene.

The ME score for each gene was scaled to fall between 0 and 1.

#### Propagating metabolomic seed labels

Label propagation was performed as stipulated by Zhou et al.^32^. The per-gene score, *f*_i_, of each node at iteration, *r*, was determined by

4 $$f_{\mathrm{i}}^{(r)} = \lambda \mathop {\sum}\limits_{{\mathrm{j}} = 1}^n {w_{{\mathrm{i}}{\mathrm{j}}}f_{\mathrm{j}}^{r - 1} + (1 - \lambda )y_{\mathrm{i}}}$$

where j is a connected node, λ is a parameter between 0 and 1 that controls the degree of propagation between a node and its neighbors, *w*_ij_, is the symmetrically normalized edge weight between node i and node j and *y*_i_ is the label of node i, which is the ME score for a particular gene. This implementation of label propagation was adapted from an implementation found at https://github.com/yamaguchiyuto/label_propagation.

Initial label values, *y*, were continuous between 0 and 1 and defined as the ME score of the corresponding gene. We used an iterative algorithm for optimizing the solution to the above equation^33^. λ was set at 0.99, as this is the parameter used in Zhou et al. and was not optimized for our data set due to limited sample size. The final scores of each of the candidate genes were ranked to generate a prioritized candidate gene list. The *homo sapiens* STRING network was downloaded from stringdb.org (v11). Each node represents a protein and an edge is present between nodes if they physically interact or share a function. Edge weight reflects the probability two proteins interact/share function based on multiple sources of evidence, including genomic context prediction (proximity in gene neighborhoods, gene fusion events, co-occurrence of proteins across species), co-expression, experimental evidence of interactions, co-citation analysis and presence in curated databases such as BioCarta and KEGG. Based on these evidences, both physical and functional interactions can be predicted to occur with high confidence (physical interactions have the “binding” signifier in the “type” column of the protein.links.full file from string-db.org). In order to determine whether physical interactions or both physical and functional interacts should be used, we analyzed the ranking of 250 causative genes from 107 patients metabolically profiled through Miller et al. when propagated on either network, and found that the ranking of the causative gene was higher when using both physical and functional STRING interactions in 90% of causative genes. Further, several causative genes in Miller et al. were not included in the network when only physical interactions were included, limiting its sensitivity. Therefore, to increase prioritization rate and sensitivity, metPropagate was applied to the complete STRING network.

#### Combining metPropagate and Exomiser rankings

We sought to create a combined metPropagate and Exomiser ranking that improved the prioritization rate while accounting for metPropagate’s bias towards genes with many metabolic first neighbors. To do this, we created a weighted additive model:

5 $$C = p \ast Exomiser + \left( {1 - p} \right) \ast metPropagate$$

Where *p* is the prior probability that a gene with a certain percentage of metabolic first neighbors is prioritized in the top 20^th^ percentile of causative genes (calculation explained below). *Exomiser* is the per-gene Exomiser-Phenotype score scaled between 0 and 1, and *metPropagate* is the per-gene metPropagate score scaled between 0 and 1. Each term of the equation is then scaled between 0 and 1 before being added together.

To calculate *p*, we first binned all 19,354 genes in STRING by the percentage of each gene’s first-degree neighbors annotated in HMDB, creating ten bins with percentage of metabolic first-degree neighbors intervals of 10% (0–10%, 10–20%, etc). Within each bin, we calculated the percentage of genes that were ranked in the top 20^th^ percentile of all 19,354 genes, calling this percentage *p*. This combined score effectively prioritizes Exomiser’s score for genes that metPropagate has a high likelihood of ranking highly due to their HMDB-neighborhood.

### Reporting summary

Further information on research design is available in the Nature Research Reporting Summary linked to this article.

## Supplementary information

Supplementary Information

Supplementary Data 1

Reporting Summary

## Data Availability

Metabolomic data from the Miller study is available through the supplement of their publication (https://www.ncbi.nlm.nih.gov/pmc/articles/PMC4626538/). Raw LC/MS data in mzML/mzData format and patient-specific Exomiser gene lists are available at https://zenodo.org/record/3774540#.Xqja9NNKg0p (10.5281/zenodo.3774540). The raw sequencing data for the patients in this study is not made available to the public or stored in third party repositories in accordance with the IRB approval (UBC IRB approval H12-00067). Details of three of the patients profiled in this study have been profiled in previous reports^2–4,6,20^. In order to facilitate reproducibility of metPropagate’s findings, all genes implicated in each TIDEX patient’s VCF are also provided at https://zenodo.org/record/3774540#.Xqja9NNKg0p. For inquiries, please contact the corresponding author or Dr. Clara van Karnebeek (c.d.vankarnebeek@amsterdamumc.nl).

## Code availability

Code for metPropagate can be found at https://github.com/emmagraham/metPropagate. There are no access restrictions. Software versions are included where referenced in the “Method” text. XCMS parameters were optimized by the IPO package, and are listed here. Peak picking was performed using the centwave algorithm within the findChromPeaks function, which had the following parameters: ppm = 15, peakdwidth = 3–80, mzdiff = 0.00325, prefilter = 3–100, noise = 1000, snthresh = 10. Peaks were grouped using the groupChromPeaks function with the sample grouping (IEM/control) as input. Retention time correction was performed using the obiwarp algorithm within the adjustRtime function using all default parameters except: gapInit = 1.2736 and gapExtend = 3.3336. Peaks were grouped again with the groupChromPeaks with the same parameters as used previously. Peaks were filled using the fillChromPeaks with all default parameters. An intensity matrix was extracted using the featureValues function (method = “medret”, value = “into”).
