# Supplementary material for: metPropagate: network-guided propagation of metabolomic information for prioritization of metabolic disease genes
Source: NPJ Genom Med. 2020 Jul 2;5:25. doi: 10.1038/s41525-020-0132-5 (PMC7331614; doi:10.1038/s41525-020-0132-5)
Supplement: Supplementary file 1 — Supplementary Information [file 41525_2020_132_MOESM1_ESM.pdf]

Supplementary Table 1

Patient number	Causative gene	Mode of inheritance	Publication ID and variant frequency (if available)	Variant information	Clinical description
1	CPT1A	Homozygous recessive, missense	PMID: 20696606, ClinVar: 65644, gnomAD: 3.246e-05	CPT1A: g.68548130G>A (p.Pro479Leu)	Collagen abnormalities (pro-collagen type Ia), osteoporosis, bone abnormalities, osteogenesis imperfecta-like, hyperphenylalaninemia, multiple congenital abnormalities (most of these likely due to her methotrextae embryopathy), recurrent coma.
2	NANS	Compound heterozygous, missense	PMID:27276562, PMID:27213289, ClinVar: 235191 , gnomAD: 0	NANS: g.100843203C>T (p.Arg237Cys), g.100840588T>C (p.Tyr188His), Transcript: ENST00000210444	Neurodevelopmental arrest, progressive epileptic encephalopathy, congenital brain abnormalities and white matter lesions
3	SCN2A	De novo, splice donor variant	PMID:27276562 and PMID:26647175, ClinVar: NA, gnomAD: 0	SCN2A: g.166188079+1 G>A, Transcript: ENST00000283256	Global developmental delay, seizures, ataxia, microcephaly, autism, abnormal CSF monoamine neurometabolite profile
4	DYRK1A	De novo, missense	PMID: 27276562, ClinVar: NA, gnomAD: 0	DYRK1A: g.38865404C>T (p.Ser346Phe), Transcript: ENST00000398960	Neurodevelopmental delay, intractable epilepsy, absence seizures, microcephaly, mild dysmorphisms, hypoglycorrhachia

5 CACNA1D	in frame deletion	ClinVar: NA, gnomAD: NA	CACNA1D: g.53842690_53842694delCCTT (p.Phe1943del)	Seizures, behavioral and psychiatric abnormalities, aggression, low CSF MTHF (folate), mild cerebral atrophy, mild ataxia, mild dysmorphism
6 CNKSR2	Homozygous from one heterozygous parent, missense	ClinVar: NA, gnomAD: NA	CNKSR2: g.21581499C>T (p. Phe464Ser), Transcript: ENST00000543067	Autonomic crises in infancy with hypertension, tachycardia, bladder retention, bowel dysmotility, frequent infections/sepsis responding to choline therapy, low acetylcholine levels, progressive cholinergic failure, alzheimers type memory loss (on treatment with Donepezil), central apneas and on BiPAP at night
7 IDS/HAL	IDS: hem-izygous, missense; HAL: compound heterozy-gous, splice donor	IDS: ClinVar: NA, gno-mAD: 0; HAL: ClinVar: NA, gnomAD: 5.99e-04	IDS: g. 148571971G>A (p.Arg294Trp ,p.Arg83Trp), Transcript: ENST00000340855, HAL: g. 96371767A>G (p.Trp537Arg, p.Trp329Arg, p.Trp68Arg), ENST00000261208 and g.96374333C>T, Transcript: ENST00000261208	Early onset global developmental delay, short stature, dysmorphisms, coarse facial features, severe behavioral disturbances, elevated keratan sulphate in urine, developmental regression, elevated glycosaminoglycans

8	CHRNA1 and DHFR	CHRNA1: de novo, missense; DHFR: de novo, missense	CHRNA1: ClinVar: NA, gnomAD: NA; DHFR: ClinVar: NA, gnomAD: NA	CHRNA1: g.175619063C>T (p.Ala167Thr/ p.Ala142Thr), Transcript: ENST00000261007; DHFR: g.79950270C>G (p.Gln13His), Transcript: ENST00000439211	Progressive global developmental delay and loss of skills, microcephaly, congenital hypotonia and wheelchair bound, dysmorphic features, severe feeding difficulties, growth retardation, demyelination on brain MRI scan, elevated lactates
9	ATP8A2	Homozygous recessive, missense	NA	ATP8A2: g.26402265G>A (p.Ala897Thr), Transcript ENST00000381655	Hypotonia, ataxia since age 18 months
10	MYO5B	Compound heterozygous, missense	ClinVar: 2.4e-03, gnomAD: 1.64e-03	MYO5B: g.47506839G>A (p.Arg344His), g.47566678G>C (p.Glu49Gln)	Intellectual disability, hyperkinetic movement disorder, sensorineural hearing loss, myopathy, malabsorption, failure to thrive, elevated urine threonine, serine and lysine, plasma amino acids suggesting lactic acidemia, elevated lactate
11	KCNQ2	KCNQ2: de novo, in frame deletion	ClinVar: NA; gnomAD: NA	KCNQ2: g.62038511_62038513delAAG (p.Phe701del), Transcript: ENST00000354587	Severe neurodegenerative disorder, failure to thrive, microcephaly, fine/gross motor delay, speech delay, intellectual disability, stereotypic behaviors, severe demyelination defect, cortical blindness, brain atrophy, dystonia, hypotonia/hypertonia, seizures, muscle weakness, progressive white matter loss, acquired microcephaly, hyperkinetic movement disorder and severe global developmental delay
